# A Rare De Novo Myoepithelial Carcinoma Ex Pleomorphic Adenoma in a Young Woman

**DOI:** 10.1155/2020/8325374

**Published:** 2020-02-26

**Authors:** Geeta Ahuja, Delaram J. Taghipour, Olubode A. Olufajo, Bonnie C. Davis, Babak Shokrani, William R. Bond

**Affiliations:** ^1^Clive O. Callender Howard-Harvard Clinical Sciences Outcomes Research Center, Department of Surgery, Howard University College of Medicine, Washington, DC, USA; ^2^Johns Hopkins Bloomberg School of Public Health and Carey Business School, Baltimore, MD, USA; ^3^Department of Radiology, Howard University College of Medicine, Washington, DC, USA; ^4^Department of Pathology, Howard University College of Medicine, Washington, DC, USA; ^5^Division of Otolaryngology/Head & Neck Surgery, Howard University College of Medicine, Washington, DC, USA

## Abstract

Carcinoma ex pleomorphic adenoma, an uncommon neoplasm of the parotid gland, accounts for less than 4% of salivary gland tumors. It arises from a benign pleomorphic adenoma presenting in the sixth to eighth decades of life. We present this as a unique account of a primary parotid gland carcinoma, arising from myoepithelial cells, without a known precursor lesion, in a 28-year-old woman. This presentation seeks to provide familiarity of an unusual presentation of an unexpected rare pathology in a young female patient and the tools utilized for an accurate diagnosis.

## 1. Introduction

Carcinoma ex pleomorphic adenoma (CXPA) is a malignant transformation that can occur in a benign pleomorphic adenoma and usually accounts for 3.6–4% of all salivary tumors and 5–15% of salivary malignancies [1–5]. CXPA typically arises in the sixth to eighth decades of life and seldom occurs in young adults [6]. The late onset is thought to be secondary to the protracted transformation into malignancy that could take an average of 20 years and up to 50 years [3]. The rate of malignant conversion is 1.5% for the first year and up to 9.5% if left untreated in 15 years [3].

Patients suffering from CXPA tend to have a poor prognosis [7]. The prognosis greatly depends on the pathological staging of CXPA; different factors, such as the extent of invasion, lymph node involvement, and metastasis location, can greatly impact the prognosis of each individual patient [1, 8]. There are various approaches to the management of CXPA particularly when the presentation is unusual.

We present a rare case of CXPA without a known precursor lesion in a 28-year-old female who went on to have a favorable treatment outcome. Written consent was obtained from the patient to publish information and images.

## 2. Case Report

**Figure 1:**
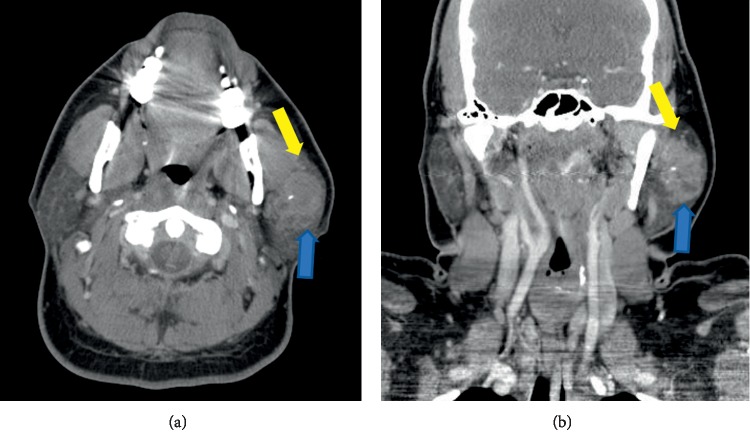
Axial (a) and coronal (b) contrast-enhanced CT images show a complex lobular mass in the left parotid gland with heterogeneous enhancement including hypodense areas of necrosis (blue arrows) and a central hyperdensity indicating a calcified component (yellow arrows).

**Figure 2:**
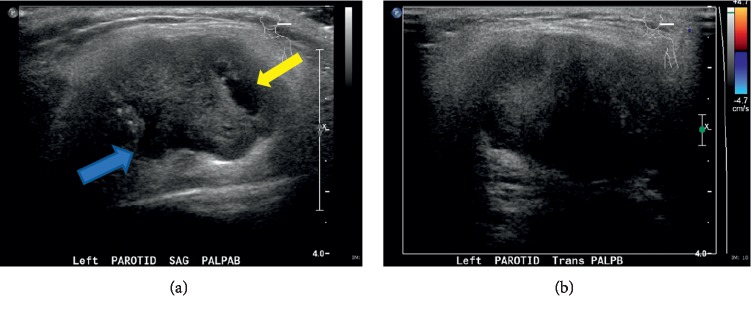
Sagittal (a) and transverse (b) ultrasound images show a well-defined lobular-shaped complex solid mass in the left parotid gland. Bright discrete echoes suggest calcifications (blue arrow; shadowing not clearly demonstrated), while an anechoic component indicates cystic fluid or necrosis (yellow arrow). Note the lack of color flow on the transverse image.

A 28-year-old woman presented with an expanding painful left-sided soft tissue swelling of the face. The mass was present for only a few months and without any associated facial nerve palsy. Examination revealed a round, well-circumscribed mass in the region of the parotid gland without overlying erythema, edema, or skin breakdown. Intraoral examination showed intact mucosa without evidence of invasion or erosion. CT scan images revealed a heterogeneous mass of the left parotid gland measuring approximately 4.0 × 2.7 × 3.9 cm with hypodense foci suggesting necrosis (Figure 1). A nonspecific level II lymph node was also visualized.  Figure 2 displays the ultrasound images of the mass.

**Figure 3:**
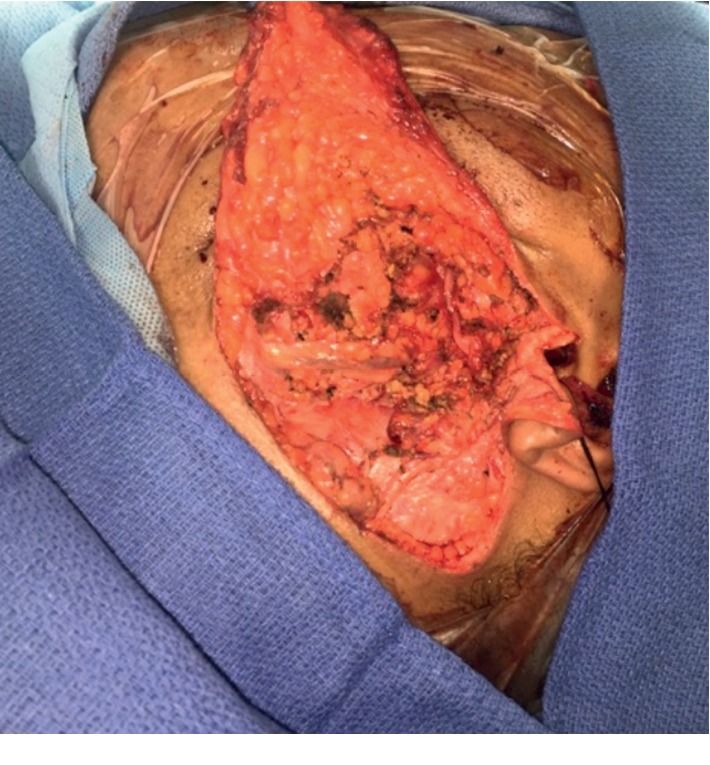
Intraoperative dissection using the Blair s-shaped incision with nerve preservation.

**Figure 4:**
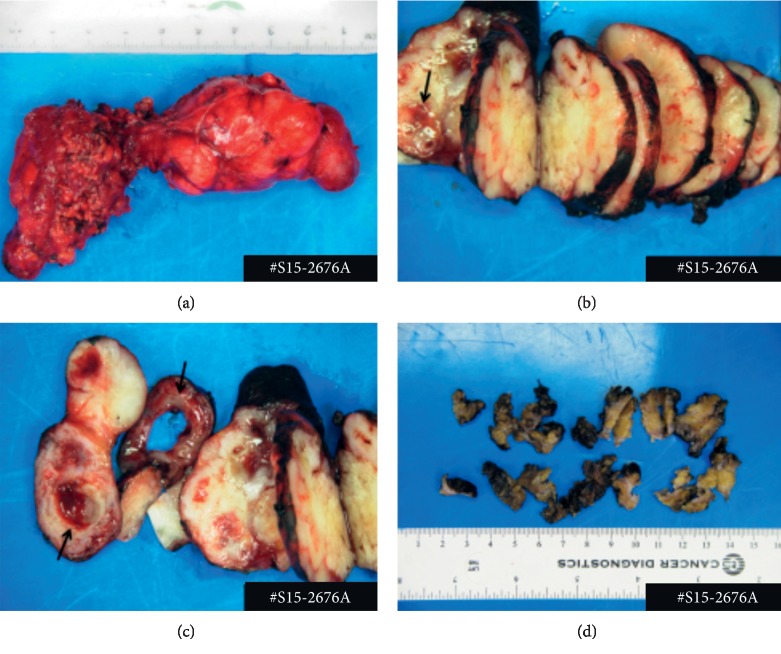
A tan/pink firm nodular mass (a, b); with areas of necrosis, hemorrhage, and cystic changes (arrows) (b, c) with attached portion of unremarkable salivary gland (d).

Fine needle aspiration and biopsy performed were consistent with a pleomorphic adenoma. At that point, the decision was made to perform a left-sided parotidectomy with intraoperative facial nerve monitoring. The Blair incision (Figure 3) was utilized to help with a deep lobe dissection. The procedure revealed a mass extending to the deep parotid lobe. Careful attention was taken to preserve all branches of the facial nerve, and the mass was surgically excised (Figure 4).

**Figure 5:**
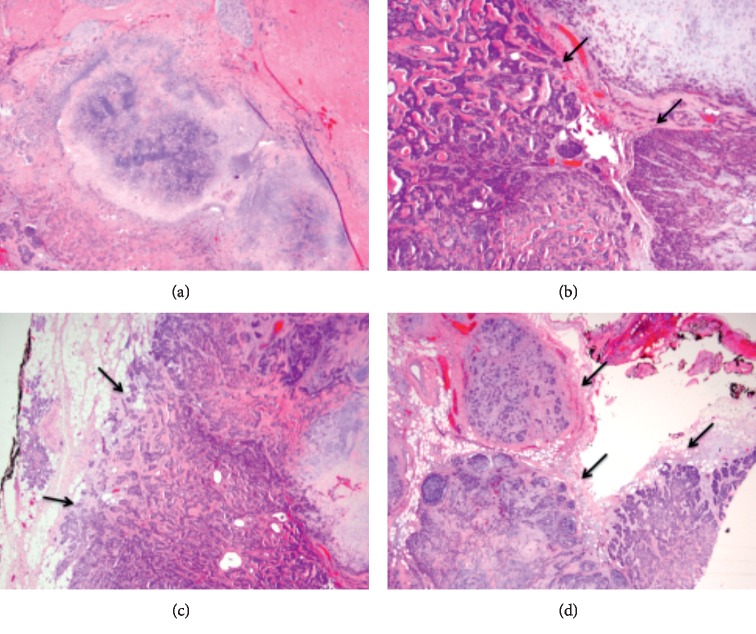
Low power microscopic sections show areas of typical pleomorphic adenoma with chondromyxoid matrix (H&E, ×40) (a), transitioning to the areas composed of cellular nests of atypical cells (arrows) (H&E, ×100) (b) with infiltration and seeding of the surrounding adipose tissue (arrows) (H&E, ×40) (c, d).

**Figure 6:**
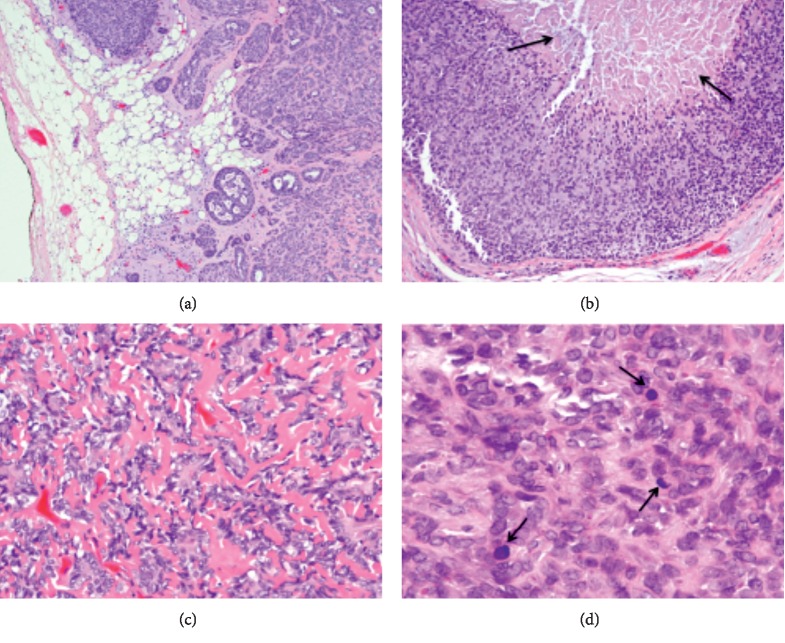
High power microscopic sections show atypical myoepithelial cells with increased mitotic activity (arrows) (3D, H&E, ×1000), hyalinized stroma (3C, H&E, ×400) and necrosis (arrows) (3B, H&E, ×200).

Histological findings displayed an intermediate grade myoepithelial carcinoma arising from a pleomorphic adenoma (Figures 5 and 6). The carcinoma showed greater than 1.5 mm invasion in to the surrounding adipose tissue (invasive type) with positive tumor-inked margins. The parotid lymph node did not show any evidence of malignancy. A pathological diagnosis of myoepithelial carcinoma ex pleomorphic adenoma was made. The patient successfully underwent adjuvant radiation therapy with a goal of 60 Gy.

Postoperatively, she experienced transient grade II House–Brackmann facial nerve dysfunction that completely resolved over the next few weeks. To date, she is asymptomatic and disease-free.

## 3. Discussion

CXPA is infrequent with a prevalence rate of 5.6 cases per 100,000 malignant neoplasms and an incidence by year of 0.17 tumors per 1 million people [1]. It occurs more commonly in females than in males and typically arises around the age of sixty to eighty [1]. The patient presented is a young female at the age of 28, who represents a rare group being treated for this disease. Typically, the mean lead up time for CXPA has been found to be nine years, with about half of the patients aware of a painless mass for below one year [6, 9]. In contrast, the CXPA in this case presented de novo and with clinical symptoms of a painful rapidly enlarging mass over a period of months, not years. Also, among a retrospective study that analyzed 151 cases of CXPA, most cases were frankly invasive high-grade malignancies [5]. Histological findings in this patient exhibited a lower grade (intermediate) myoepithelial carcinoma, again contrasting the typical findings of CXPA. The combination of all these features makes this an exceptional case worthy of reporting.

Several epidemiological variations in the presentation of CXPA have been observed. For example, in Switzerland, CXPA was found to make up 14% of all primary parotid malignant neoplasms over 20 years [10]. In the United States, the reported prevalence was 12% of all primary parotid malignant neoplasms [11]. In terms of the racial distribution of CXPA in the United States, the incidence data shows a rate of 0.77 persons per million in Blacks, 0.71 persons per million in Whites, and 0.34 persons per million among other races [12].

The exact etiology for transformation to CXPA is not well understood. Various studies suggest radiation, genetic instability within the tumor, and increased expression of the cytoplasm of p16 protein by promotor methylation of the p16 gene may all contribute [13, 14]. A molecular study suggests a multistep model with the progressive loss of heterozygosity at chromosomal arms 8q, then 12q, and finally 17p [2, 15]. Genes involved in tumor suppression, cell cycle control, growth, etc., are also involved in the development of CXPA [7]. For example, the *p53* gene has been associated with the development of malignant CXPA; point mutation of p53 was reported originally by Righi et al. [16]. Righi et al. found positive expression of the p53 protein and mutation of p53 in four instances of CXPA [16]. Additionally, CXPA cases can be divided into those with only epithelial (luminal) malignancy and those with myoepithelial (nonluminal) malignancy [7]. Myoepithelial cells have been shown to provide fuel for tumor progression [17]. It is hoped that these markers could be used to provide helpful hints in the future diagnosis and management of CXPA and lead to a better prognosis for affected patients [7].

CXPA normally presents as a firm mass on the parotid gland [7]. Patients may only become aware of the tumor when they notice rapid enlargement and pain and are otherwise asymptomatic [6]. A patient diagnosed with noninvasive or minimally invasive CXPA has a much better prognosis than a patient with invasive CXPA diagnosis [8]. The tumor size and grade are also important factors in determining the prognosis [8]. Besides all these factors, early accurate diagnosis and appropriate surgical and radiological management of CXPA are essential in improving the outcome of this disease [8].

Preoperative diagnosis of CXPA can be challenging for clinicians and pathologists [7]. When there is a high clinical or radiological suspicion, pathological confirmation is sought [7]. Fine needle aspiration is frequently used to diagnose CXPA, although its sensitivity is relatively low [7]. An earlier study in 1999 showed a relatively high rate of sensitivity, in which 54% of patients with CXPA were diagnosed before operation with fine needle sampling [18]. However, in a recent study conducted in 2008, 44% of CXPA cases were found to be malignant by fine needle aspiration, and a 2005 study found the number to be even lower, at only 29% [10, 19]. When used alone, the preoperative diagnostic methods available for CXPA lack veracity. Therefore, a combination of diagnostic tools including radiological and pathological tests is required in order to best make an accurate preoperative diagnosis [20].

The definitive treatment for CXPA usually involves ablative surgery, which may be followed with reconstructive surgery [6, 19]. The specific surgical approach varies depending on the site of the CXPA. Because CXPA tends to affect the parotid gland, surgery typically involves parotidectomy [6, 19]. For CXPA in the lacrimal gland, lateral rhinotomy and medial maxillectomy is recommended [21]. Tracheal CXPA requires a segmental tracheal resection and a suprahyoid release [22]. For high-grade disease, indeterminate resection adequacy and lymph node and perineural invasion, adjuvant radiotherapy is usually employed [9]. Combination of radiotherapy and chemotherapy is used in selected cases [9, 19].

The reported 5-year survival of CXPA has been found to vary. A 5-year survival rate of 37% among 73 patients and 44% among 28 patients was found among studies conducted in 2001 and 2005, respectively [6, 19]. Another study by Zbaren et al. found the 5-year survival rate to be much higher, at 75% among 24 patients [10]. This higher survival rate could be due to the greater amount of intracapsular CXPA in the study since intracapsular CXPA can act similar to a benign pleomorphic adenoma and have a better prognosis [9].

## 4. Conclusion

CXPA remains a rare disease that is difficult to diagnose both clinically and pathologically. Because of the significant clinical implications associated with the late diagnosis of the disease, the ongoing efforts to identify better diagnostic modalities are critical. This rare case of CXPA presented is thus vital to furthering the current understanding of the condition, as it represents a novel presentation and can serve as an exemplar for future diagnoses. Further research and investigation on CXPA are needed to help lead to earlier diagnoses and treatments for patients.
